# Assessing peri-operative antibiotic administration practices amongst urologic surgeons performing holmium laser enucleation of the prostate worldwide

**DOI:** 10.1007/s00345-025-05535-2

**Published:** 2025-03-13

**Authors:** Seyed Mohammad Mohaghegh Poor, Hafsa Asif, Darion Denis-Diaz, Eric Riedinger, Tasha Posid, Maxwell Newton, Michael Sourial, Mark Assmus, Amy Krambeck, Bodo Knudsen, Matthew Lee

**Affiliations:** 1 Division of Urology, Department of Surgery, The University of Vermont, Burlington, VT USA; 2 Wexner Medical Center, Department of Urology, The Ohio State University, Columbus, OH USA; 3 Department of Urology, The University of Tennessee Knoxville, Knoxville, TN USA; 4 University of Calgary, Southern Alberta Institute of Urology, Calgary, AB Canada; 5 Department of Urology, Northwestern University, Chicago, IL USA

**Keywords:** Benign Prostatic Hyperplasia, HoLEP, Antibiotic Stewardship

## Abstract

**Purpose:**

Holmium Laser Enucleation of the Prostate (HoLEP) is a size-independent surgical treatment for benign prostatic hypertrophy. There is currently a lack of data on peri-operative antibiotic prescribing patterns for HoLEP and, thus, no consensus on optimal practices. This study aims to assess peri-operative antibiotic prescribing practices for HoLEP.

**Methods:**

Members of the Endourological Society (EUS) were invited by e-mail to complete a REDCap survey. The survey inquired about surgeons’ practice setting, training, surgical volume, antibiotic prescribing practices and explored different factors that might affect antibiotic choice and duration. A p-value of < 0.05 was determined to be statistically significant.

**Results:**

A total of 70 Urologists (66 male, 4 female) reported that they performed an average of 108 HoLEPs per year with a mean clinical experience of 11 years. In the case of a negative pre-operative urine culture with a patient who is not catheterized/intermittently self-catheterizing (C/ISC), 96% of urologists would only give a single peri-operative dose of antibiotic. If the patient is C/ISC then 49% of Urologists would give more than a single dose of peri-operative antibiotic when the urine culture is negative. If the pre-operative urine culture is negative, 39% of surgeons would prescribe post-operative antibiotics even when the patient is not C/ISC and this increased to 64% if the patient is C/ISC. The most common factors urologists considered when prescribing antibiotic prophylaxis/therapy were positive urine culture, catheterization status, and a history of recurrent UTIs. Non-academic urologists administered post-operative prophylaxis more often (*p* < 0.05) and urologists with more experience treated a positive urine culture for a shorter period.

**Conclusion:**

There is significant variability for peri-operative antibiotic prescribing practices prior to HoLEP. In general, more antibiotics are prescribed if the patient has a history of C/ISC or infection. Further clinical studies are needed to identify optimal antibiotic prescribing protocols prior to HoLEP.

**Supplementary Information:**

The online version contains supplementary material available at 10.1007/s00345-025-05535-2.

## Introduction

Holmium Laser Enucleation of the Prostate is a size-independent treatment option for lower urinary tract symptoms caused by benign prostatic hypertrophy (BPH) per the American Urological Association (AUA) guidelines [1]. Despite having been described in 1997, HoLEP has not been widely adopted and accounts for less than 5% of all BPH procedures performed in a recent review. The main reason for the limited uptake is thought to be its steep learning curve [2]. As interest in HoLEP increases, clear guidelines for peri-operative management of patients undergoing this procedure are essential. An important area is post-operative infection prevention. Urinary tract infection (UTI) and sepsis are concerning complications. To minimize the risk of these complications, the AUA suggests a single dose of antibiotic prophylaxis to reduce the risk of sepsis and UTI in patients undergoing transurethral procedures [3]. However, there is a lack of consensus on how to manage patients with indwelling catheters and if these patients require additional prophylaxis.

There have been few clinical trials investigating the role of different antibiotic prophylaxis regimens prior to HoLEP. Prior literature supports the use of antibiotic prophylaxis to reduce the risk of infectious complications [4]. However, there is contradictory evidence whether extending the duration of antibiotic prophylaxis for transurethral prostate surgery is beneficial in reducing the risk of post-operative infection [5–7]. Thus, the aim of this study was to assess surgeons’ preferences regarding the type and duration of peri-operative antibiotic practices around HoLEP. In addition, we investigated which factors might influence surgeon antibiotic prescribing practices and how various surgeon characteristics might affect those choices.

## Methods

Members of the Endourological Society (*n* = 1310) [8] were invited by e-mail to complete a REDCap survey. This study was deemed Institutional Review Board (IRB) exempt by the Ohio State University IRB as no patient/participant identifying information was recorded. The survey was designed in-house at The Ohio State University by the study authors and consisted of 18 questions about surgeons’ practice setting, training, surgical volume, and antibiotic prescribing practices for various clinical scenarios. The complete survey questionnaire is attached in Appendix 1. The survey was sent out electronically a total of 3 times with a two-week interval between each email, until no further responses were obtained. Data from REDCap was tabulated and analyzed using SAS v9.4. Chi-square tests were performed for categorical factors and logistic regressions were performed for continuous variables to test for associations with antibiotic usage. A p-value of < 0.05 was determined to be statistically significant.

## Results

### Demographics

Of the 1310 members surveyed, 70 responded (5.3%, *n* = 66 male, 4 female). Respondents performed an average of 108 HoLEPs per year (range = 1 to 750) with a mean clinical experience of 11 years. Results for surgeon country of practice and practice setting are provided in Supplementary Tables 1 and Fig. 1, respectively. Most surgeons were from the United States and worked at an academic medical center (36% and 69%, respectively). HoLEP surgeons obtained the skill though fellowship (39%), self-training (33%), course-based training (17%) or other methods (11%). Other methods included: HoLEP training in residency, visiting a HoLEP expert or learning from a senior colleague.

### Antibiotic prophylaxis practice patterns

For a patient with a negative preoperative urine culture, who was not catheterized/ intermittently self-catheterizing (C/ISC), 95.7% of urologists would only give a single-dose, peri-operative, prophylactic antibiotic and no additional pre-operative prophylaxis (Table 1). Practice setting was the only factor associated with additional antibiotic prophylaxis for this scenario. Private practice surgeons prescribed more post-operative antibiotics than Academic or Hospital-employed surgeons: 75.0% vs. 64.6% vs. 11.0%, respectively (*p* = 0.015, Supplementary Table 2).

**Table 1:** Urologist peri-operative antibiotic practices for different clinical situations. Percentages are shown in parentheses and calculated as a percentage of peri-operative vs. post-operative total columns. Ucx = Urine culture. PPX = prophylaxis. ABX = antibiotics. N/a = Not assessed

Clinical Situation	Peri-Operative Antibiotics (%)							Post-Operative Antibiotics (%)				
	Single Dose PPX	3d Pre-Op ABX	5d Pre-Op ABX	7d Pre-Op ABX	10d Pre-Op ABX	14d Pre-Op ABX	Other Pre-Op	3d Post-Op ABX	5d Post-Op ABX	7d Post-Op ABX	Other Post-op	*p*-value
Negative Ucx, No C/ISC	67 (95.7)	0 (0.0)	1 (1.4)	1 (1.4)	0 (0.0)	0 (0.0)	1 (1.4)	6 (22.2)	12 (44.4)	8 (29.6)	1 (3.7)	< 0.0001
Negative Ucx, +C/ISC	36 (51.4)	20 (28.6)	7 (10.0)	5 (7.1)	0 (0.0)	0 (0.0)	2 (2.9)	11 (25.0)	17 (38.6)	14 (31.8)	2 (4.6)	
Positive Ucx	0 (0.0)	13 (18.8)	16 (23.1)	30 (43.5)	5 (7.3)	3 (4.4)	2 (2.9)	N/a				
Mixed Flora result on Ucx	40 (58.0)	7 (10.1)	11 (15.9)	9 (13.0)	1 (1.4)	1 (1.4)	0 (0.0)	N/a				

For a patient with a negative pre-operative urine culture and is C/ISC, 51.4% of urologists would only give a single peri-operative dose (Table 1). However, 48.6% of urologists would give additional prophylaxis; specifically, 28.6% (*n* = 20/70) of urologists would prescribe 3 days of pre-operative prophylaxis and 24.2% of the cohort (*n* = 17/70) would prescribe 5 days of post-operative antibiotics. Five days was the most common post-operative duration, accounting for 38.6% of urologists who prescribe post-operative antibiotics (Table 1). There was a significant association between practice setting and prescription of post-operative antibiotics prophylaxis for this situation; with 92% of private practice urologists vs. 66% of academic urologists and 22% of hospital-employed urologists prescribing post-operative antibiotics (*p* = 0.006, Supplementary Table 2). For pre-operative prophylaxis in this situation, 66.7% private practice urologists vs. 50% of academic urologists and 22.2% of hospital-employed urologists prescribed pre-operative prophylactic antibiotics, but this difference was not statistically significant (Supplementary Table 2).

C/ISC status influenced peri-operative antibiotic choice and duration. For example, when the patient has a negative pre-operative urine culture but is C/ISC, 62.8% (*n* = 44/70) of urologists will prescribe post-operative antibiotic prophylaxis vs. 39% (*n* = 27/70) when the patient is not C/ISC (*p* < 0.0001, Table 1). Furthermore, more surgeons opted for broader coverage for patients who are C/ISC (Table 2). For example, for patients with a negative pre-operative urine culture and *no* history of C/ISC, 31.4% and 12.9% of urologists prescribed cefazolin and ampicillin + gentamicin or ampicillin/sulbactam + gentamicin, respectively, for perioperative prophylaxis. For patients with a negative pre-operative urine culture and *positive* history of C/ISC, the percentage of urologists prescribing cefazolin mono-prophylaxis decreased to 20.3% and the percentage prescribing ampicillin + gentamicin or ampicillin/sulbactam + gentamicin increased to 20.3% (*p* < 0.0001, Table 2). Additionally, the percentage of urologists prescribing trimethoprim-sulfamethoxazole or ciprofloxacin post-operatively increased for a situation where the patient was C/ISC (*p* < 0.0001, Table 2).

**Table 2:** Antibiotic prophylaxis choices for various clinical situations. Ucx = Urine culture. C/ISC = catheterized/ intermittent straight catheterization. (+) = positive/present. (-) = negative/no. N/a = not applicable

Pre-Op Single-Dose Antibiotic Prophylaxis Choice (%)										
Clinical Situation	Cefazolin/ Cephalexin	Augmentin	Ceftriaxone	Ampicillin + Gentamycin	Ampicillin/ Sulbactam + Gentamycin	Macrobid	TMP/SMX	Ciprofloxacin	Other	*p*-value
**-Ucx**, **-C/ISC**	22 (31.4)	N/a	20 (28.6)	7 (10.0)	2 (2.9)	N/a	0 (0.0)	4 (5.7)	15 (21.4)	< 0.0001
**-Ucx**,** +C/ISC**	14 (20.3)	N/a	20 (29.0)	10 (14.5)	4 (5.8)	N/a	0 (0.0)	4 (5.8)	17 (24.6)	
**Pre-Op Oral Antibiotic Prophylaxis Choice (%)**										
**-Ucx**, **-C/ISC**	0 (0.0)	0 (0.0)	0 (0.0)	0 (0.0)	0 (0.0)	1 (33.3)	2 (66.7)	0 (0.0)	0 (0.0)	N/a
**-Ucx**,** +C/ISC**	2 (5.9)	0 (0.0)	3 (8.8)	0 (0.0)	0 (0.0)	9 (26.5)	7 (20.6)	11 (32.4)	2 (5.9)	
**Post-Op Oral Antibiotic Prophylaxis Choice (%)**										
**-Ucx**, **-C/ISC**	7 (25.9)	2 (7.4)	N/a	N/a	N/a	4 (14.8)	3 (11.1)	6 (22.2)	5 (18.5)	< 0.0001
**-Ucx**,** +C/ISC**	7 (16.3)	2 (4.7)	N/a	N/a	N/a	6 (14.0)	10 (23.3)	13 (30.2)	5 (11.6)	

For patients with a positive preoperative urine culture, all urologists treated the bacteria with antibiotic therapy prior to HoLEP and treatment courses ranged from three days (18.8%) to 14-days (4.4%) of antibiotics (Table 1). Seven days was the most common treatment duration, accounting for 43.5% of surgeons. Urologists with more years of experience utilized a shorter duration of antibiotic treatment. Our findings showed for each additional year of urologist experience, antibiotic treatment duration decreased by 0.078 days (Estimate: -0.078 day, *p* = 0.005).

If the pre-operative urine culture resulted in “Mixed Flora”, 34% (*n* = 24/70) of surgeons will request further speciation and 42% (*n* = 29/69) will treat the mixed flora result preoperatively even if further speciation is not possible. Surgeons who learned HoLEP from a course (*p* = 0.04) and private practice surgeons (*p* = 0.01) were associated with requesting further speciation of mixed flora urine cultures. No surgeon characteristics were associated with preoperative treatment of a mixed flora urine culture. Treatment of a mixed flora result was widely variable; 58.0% (*n* = 40/69) of urologists would only use single-dose peri-operative prophylaxis. Of the 42% who would treat preoperatively, five days was the most common treatment duration (37.9%, *n* = 11/29, Table 1).

Urologists considered a variety of factors when choosing the type and duration of antibiotic therapy and prophylaxis around the time of HoLEP. The top three factors that urologists considered when prescribing antibiotic prophylaxis and therapy were: positive preoperative urine culture (97%), catheterization status (84%), and a history of recurrent UTIs (73%, Supplementary Table 3).

## Discussion

There is a lack of consensus on best practices for peri-operative antibiotic prophylaxis prior to HoLEP [3, 9]. The best prophylactic regimens balance the risk of serious peri-operative infection, prioritizes antibiotic stewardship, prevent adverse drug reactions, and decrease unnecessary healthcare costs. In general, our study identified that urologists prescribe more antibiotics if a patient is catheterized or has a history of infections. Furthermore, there is significant variability in antibiotic prophylaxis prescription patterns.

In our study, most urologists (95.7%) will only give a single dose of peri-operative antibiotics to a patient with a negative pre-operative urine culture and no history of C/ISC. Reported rates of UTI and fever after monopolar TURP, bipolar TURP, and HoLEP are 4.1%, 2.6%, and 0.9%, respectively. Rates of sepsis for these transurethral surgeries range between 0 and 0.1% [10]. Therefore, given the rarity of sepsis, some authors have even proposed that no antibiotic prophylaxis be given prior to transurethral surgery for select patients [7, 11, 12]. Indeed, in one prospective, randomized controlled trial that excluded patients with preoperative pyuria (> 100 WBC/mL) or an indwelling catheter, the authors found that omission of all antibiotic prophylaxis did not increase the risk of post-operative fever (*p* = 0.8) [7]. Given the minimal risk of antibiotic resistance with a single peri-operative prophylaxis dose and the risk of morbidity from UTI or urinary sepsis post transurethral surgery, we do not advocate for omission of prophylaxis. However, studies like this push the envelope of antibiotic stewardship and should make us re-examine our antibiotic prescribing practices. Indeed, we identified that 75% and 35% of urologists in private practice and academic practice, respectively, prescribe additional post-operative antibiotics even for a patient with a negative preoperative urine culture and no C/ISC. Results from the randomized controlled trial that excluded *all* antibiotic prophylaxis without additional infections complications suggest that these additional post-operative antibiotics could likely be safely omitted [7]. 

Urologists prescribed more and broader coverage pre- and post-operative antibiotic prophylaxis for catheterized patients, even when the preoperative urine culture was negative, suggesting that surgeons were more concerned about infectious complications for patients who are C/ISC. Indeed, in one study, 33% of catheterized patients with sterile urine developed a post-operative UTI vs. 14% of non-catheterized patients [11]. Fortunately, antibiotic prophylaxis reduced the risk of post-operative UTI for these catheterized patients to 14%. However, the ideal duration of prophylaxis for these catheterized patients is unknown. In one retrospective study analyzing infectious complications post-HoLEP, the authors reported outcomes for 90 patients and identified that there was no difference in post-operative infectious complications for antibiotic prophylaxis courses that were $\:\le\:$ 2 days vs. those that were $\:\ge\:$ 3 days [13] This study did not compare the incidence of post-operative infectious complications between patients who were and were not catheterized. In another retrospective analysis of 222 patients undergoing TURP, the authors performed an economic analysis studying the effects of antibiotic prophylaxis and found that using broad-spectrum antibiotics or longer courses of antibiotic prophylaxis did not reduce the incidence of UTI, but did increase healthcare costs [14]. The authors did not identify an association between an indwelling catheter and increased length of stay. However, the authors did not specifically analyze if catheterization status was a risk factor for infectious complications. Since only three patients developed UTI requiring hospitalization, the authors recommended only using 24 h of prophylaxis. Although our study did not examine rates of postoperative infection after HoLEP with varying durations of antibiotic prophylaxis, we include these studies as their results suggest that longer courses of antibiotics may not be more effective than shorter courses at reducing post-operative infectious complications. Furthermore, these studies did not comment on the effect of prophylaxis for patients who are C/ISC.

A known risk factor for post-operative infection is a pre-operative positive urine culture [15–18]. Almost all participants (97%) of our study considered preoperative urine culture results when determining the type and duration of antibiotic therapy prior to HoLEP. However, there was a lack of consensus on treatment duration. Of note, urologists with more experience treated a positive culture with a shorter duration. Given the lack of strong data supporting the extended duration of peri-operative antibiotic prophylaxis, it is possible a shorter course may be more appropriate for antibiotic stewardship. Our study did not specifically ask if the presence of certain bacterial species would affect antibiotic choice or duration. To our knowledge, this has not been studied in the context of the HoLEP. However, in the TURP literature, one study found that certain bacterial species including Enterococcus Faecium, Klebsiella Pneumoniae, and Pseudomonas Aeruginosa, were associated with higher risk of post-operative infection [18]. 

There is also limited evidence to guide the management of urine cultures with mixed flora. These results are often dismissed as sample contamination [19]. Studies of males and females seen in urology clinics reported that 46–68% of urine culture results had a mixed flora [19, 20]. In our study, 58% of urologists reported they do not treat these results preoperatively. Fellowship trained HoLEP surgeons were less likely to treat a mixed urinary flora culture result preoperatively. While there may be value in obtaining further speciation to understand the sensitivity pattern of the mixed urinary flora results and to minimize the usage of broad-spectrum antibiotics, only 34% of Urologists in our study requested this. Presuming that surgeons who only use single-dose peri-operative prophylaxis for patients with a pre-operative urine culture with mixed flora or those who do not request further speciation, do not have increased infectious complications compared to surgeons who prescribe additional pre-operative prophylaxis or request further speciation, this suggests that pre-operative prophylaxis and further speciation may be unnecessary for this scenario.

Our survey results identified that urologists considered a positive preoperative urine culture, catheterization status, and a history of recurrent UTIs as the most concerning patient characteristics when choosing antibiotic choice and duration for therapy or prophylaxis. Other risk factors that have been reported to increase risk of post-operative UTI after HoLEP include: increasing BMI, frailty, and positive preoperative urine culture [21]. Additionally, one study identified that a history of diabetes and increased operative time were associated with UTI after various prostate surgery including: TURP, HoLEP and Photovaporization of Prostate [22]. 

Limitations of our study include that it is survey based, which introduces bias. In addition, there was a limited response rate of 5.3%. This is lower than some other recent survey-based studies of the endourological society [23]. However, it is reasonable to assume that not all EUS members perform HoLEP since it only accounts for 5% of all BPH surgeries. The true response rate is likely much higher than 5.3% adjusted for the denominator [2]. Another limitation is that we did not inquire about other energy forms to perform endoscopic enucleation such as bipolar electrocautery, thulium fiber laser, greenlight laser or thulium: YAG laser. Including these additional enucleation-based modalities may have improved our response rates. Nevertheless, the survey was sent electronically three times with a two-week period between survey requests, and we did not have any increase in response rate after the third and final attempt. Another limitation is that our results may not reflect the practices of less experienced or lower volume surgeons since the average practice experience and HoLEP volume of responding surgeons was 11 years and 108 cases per year.

Finally, another limitation of our study is that during our initial survey design we did not consider that urologists may prescribe post-operative antibiotics for patients with a mixed flora urine culture result or a positive pre-operative urine culture. Thus, our survey only queried about pre-operative practice patterns for these situations and does not yield insights on post-operative practice patterns. This can be an area of future study. Additional future efforts should focus on assessing the safety of different antibiotic prophylaxis regimens prior to HoLEP and some efforts are already underway [24, 25]. 

## Conclusion

There is significant heterogeneity for peri-operative antibiotic prophylaxis at the time of HoLEP. More antibiotics were prescribed if a patient was catheterized or had a positive pre-operative urine culture. The duration of antibiotic prophylaxis prescribed for patients who are C/ISC had wide variation. Clinical studies are needed to assess the best protocols for antibiotic prophylaxis at the time of HoLEP and other endoscopic enucleation-based techniques, particularly for patients who are catheterized, have a positive preoperative urine culture or a history of recurrent UTI. Furthermore, it would be interesting to see if antibiotic prophylaxis patterns differ for enucleation-based surgeries vs. TURP. Lastly, consensus-based guidelines tailored to HoLEP and other endoscopic enucleation-based procedures are needed to raise awareness of this issue and allow clinicians to minimize infectious complications and practice antibiotic stewardship.

## Electronic supplementary material

Below is the link to the electronic supplementary material.

Supplementary Material 1

## Data Availability

No datasets were generated or analysed during the current study.
