# Supplementary material for: Assessing peri-operative antibiotic administration practices amongst urologic surgeons performing holmium laser enucleation of the prostate worldwide
Source: World J Urol. 2025 Mar 13;43(1):169. doi: 10.1007/s00345-025-05535-2 (PMC11906547; doi:10.1007/s00345-025-05535-2)
Supplement: Supplementary file 1 — Supplementary Material 1 [file 345_2025_5535_MOESM1_ESM.docx]

**Supplementary Table 1.** Countries represented by survey respondents

| **Country** | **HoLEP Surgeons (N)** | **Percent** |
| --- | --- | --- |
| United States | 25 | 35.7% |
| Canada | 4 | 5.7% |
| Turkey | 4 | 5.7% |
| Greece | 3 | 4.3% |
| India | 3 | 4.3% |
| Italy | 3 | 4.3% |
| Spain | 3 | 4.3% |
| United Kingdom | 3 | 4.3% |
| Begium | 2 | 2.9% |
| Germany | 2 | 2.9% |
| Israel | 2 | 2.9% |
| Argentina | 1 | 1.4% |
| Australia | 1 | 1.4% |
| Brazil | 1 | 1.4% |
| Chile | 1 | 1.4% |
| Egypt | 1 | 1.4% |
| France | 1 | 1.4% |
| Iceland | 1 | 1.4% |
| Japan | 1 | 1.4% |
| Lithuania | 1 | 1.4% |
| New Zealand | 1 | 1.4% |
| Pakistan | 1 | 1.4% |
| Philipines | 1 | 1.4% |
| Poland | 1 | 1.4% |
| South Africa | 1 | 1.4% |
| Switzerland | 1 | 1.4% |
| Ukraine | 1 | 1.4% |

**Supplementary Figure 1.** Practice setting of survey respondents


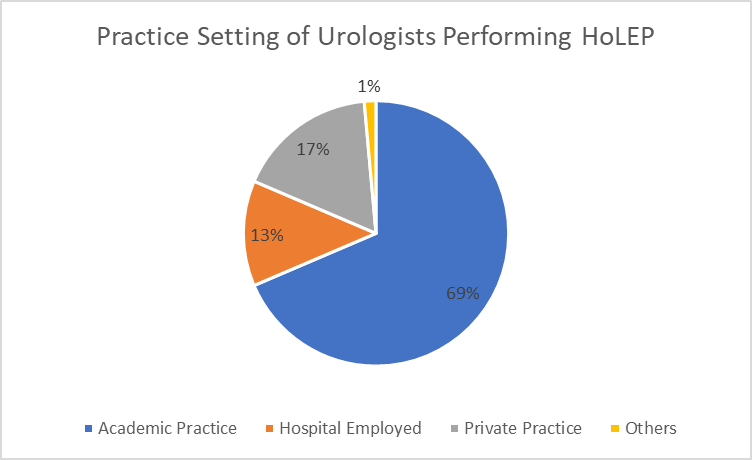


**Supplementary Table 2. Perioperative antibiotic practices for various clinical scenarios by practice setting. Percentages are calculated by practice setting e.g. 95.8% of academic urologists prescribe single-dose peri-operative prophylaxis. Abx = antibiotics. Ucx = Urine culture. C/ISC = catheterized/intermittent straight catheterization. (+) = positive/present. (-) = negative/no**

| **Practice Setting** | **Single-dose peri-op Abx, -Ucx, -C/ISC** | **p-value** | **Postop Abx,**  **-Ucx, -C/ISC** | **p-value** | **Preop Abx,**  **-Ucx, +C/ISC** | **p-value** | **Postop Abx, -Ucx, +C/ISC** | **p-value** |
| --- | --- | --- | --- | --- | --- | --- | --- | --- |
| **Academic** | **46 (95.8)** | **0.8** | **31 (64.6)** | **0.0154** | **24 (50.0)** | **0.17** | **31 (66.0)** | **0.006** |
| **Hospital-Employed** | **9 (100.0)** |  | **1 (11.1)** |  | **2 (22.2)** |  | **2 (22.2)** |  |
| **Private** | **12 (100.0)** |  | **9 (75.0)** |  | **8 (66.7)** |  | **11 (91.7)** |  |
| **Other** | **1 (100.0)** |  | **0 (0.0)** |  | **0 (0.0)** |  | **0 (0.0)** |  |

**Supplementary Table 3**. Factors Urologists consider when choosing antibiotic therapy or prophylaxis

| **Factors** | **n (%)** |
| --- | --- |
| Positive Urine Culture | 68 (97.1) |
| Catheterization Status | 59 (84.3) |
| History of Recurrent UTI | 51 (72.9) |
| History of Prostatitis | 32 (45.7) |
| Patient's Age | 22 (31.4) |
| Duration of the Procedure | 11 (15.7) |
| Bladder Stone | 17 (24.3) |
| Size of the Prostate | 2 (2.9) |
| Other Factors | 2 (2.9) |
| Degree of Hematuria | 1 (1.4) |
